# Dietary fiber and probiotics based on gut microbiota targeting for functional constipation in children with cerebral palsy

**DOI:** 10.3389/fped.2022.1001789

**Published:** 2022-10-06

**Authors:** Congfu Huang, Jinli Lyu, Chunuo Chu, Lan Ge, Yuanping Peng, Zhenyu Yang, Shenghua Xiong, Bin Wu, Xiao Chen, Xiaowei Zhang

**Affiliations:** ^1^Department of Pediatrics, Longgang District Maternity and Child Healthcare Hospital, Shenzhen, China; ^2^Department of Obstetrics and Gynecology, Peking University Shenzhen Hospital, Shenzhen, China; ^3^Shenzhen Middle School, Shenzhen, China; ^4^Department of Nutrition, BGI Nutrition Precision Co., Ltd., Shenzhen, China; ^5^The Outpatient Department, Longgang District Social Welfare Center, Shenzhen, China; ^6^Department of Microbial Research, WeHealthGene Institute, Joint Laboratory of Micro-Ecology and Children's Health, Shenzhen Children's Hospital, Shenzhen WeHealthGene Co., Ltd., Shenzhen, China

**Keywords:** gut microbiota, dietary fiber, probiotics, cerebral palsy, functional constipation

## Abstract

Gastrointestinal (GI) disorders are very common among children with cerebral palsy. Gut microbiota has been confirmed to maintain normal GI physiological function and further contributed to cerebral palsy through the gut-brain axis. Our study was to investigate the effect of dietary fiber combined with probiotics on functional constipated children with cerebral palsy. In total, 35 patient children were enrolled and divided into general diet group (*n* = 14) and liquid diet group (*n* = 21). All the participants received Compound Dietary Fiber (CDF) for 1 month and lactic acid-producing and butyric acid-producing probiotics for 6 months. After a 1-month intervention, the frequency of spontaneous and manual defecation, and Bristol score were all significantly improved (*P* < 0.001). The α-diversity of the gut microbiota was significantly increased after a 1-month intervention (*P* < 0.05), with a higher abundance of butyric acid-producing bacteria and a lower abundance of opportunistic pathogens (*P* < 0.05, FDR < 0.05). However, the impersistent effect of the 6-month intervention suggested the insufficient impact of intaking probiotics alone and the short duration of CDF intervention. Moreover, although the intervention had affected the constipation symptoms equally in cerebral palsy children with a general diet and liquid diet, the general diet group showed a greater and more durable change in gut microbiota and clinical phenotypes after intervention than the liquid diet group, which indicated that longer intervention time should be considered for liquid diet children. This study not only illustrated that supplementation of dietary fiber combined with probiotics can improve functional constipation in children with cerebral palsy, but also provides guidance for optimal intervention strategy for future studies, which will further benefit cerebral palsy children.

**Clinical trial registration:**

http://www.chictr.org.cn/showproj.aspx?proj=46902, identifier: ChiCTR1900028257.

## Introduction

Gastrointestinal (GI) disorders are very common among children with cerebral palsy, among which the most common one is constipation. The incidence of constipation in children with cerebral palsy reaches 26–74%, which is much higher than it in healthy children (1–3). It is often accompanied by other symptoms, such as boating, vomiting, gastroesophageal refluxing, and GI bleeding (4). Recurrent constipation in children with cerebral palsy is a kind of functional constipation that is defined as the disorder of brain-gut axis interaction (5). Gut microbiota is crucial to maintain the normal physiological function of the GI tract. The imbalance of gut microbiota will result in GI diseases, including constipation, which may further contribute to the occurrence of cerebral palsy. Adjusting the dietary uptake has been confirmed to be an efficient way to improve GI function by modulating the gut microbiota.

Dietary fiber is a kind of carbohydrate that can be neither digested nor absorbed in the small intestine, and will be fermented by the bacteria in the GI tract (6). Dietary fiber provides a nutrient source for gut microbiota, maintaining the integrity of the intestinal barrier. It has been reported that dietary fiber deficiency can force gut microbiota to use host-secreted mucus glycoproteins as an alternative nutrient source, leading to erosion of the colonic mucus barrier with greater epithelial access and predisposition to colitis (7). In addition, it can stimulate the production of short-chain fatty acids (SCFAs) by modulating the gut microbiota, which will regulate the immune response of the host (8). Psyllium husk is one kind of dietary fiber, consisting of highly branched and gel-forming arabinoxylan, which can be utilized by many members of the gut microbiota as an energy source. Moreover, it is capable of retaining water in the small intestine, thereby increasing water flow into the ascending colon (9). It has been reported that psyllium husk was successfully used in the symptomatic therapy of constipation (10–12). It can improve the defecation frequency and the stool consistency of patients with chronic constipation (11). In addition, it has been listed as recommended dietary fiber for the treatment of chronic constipation by the World Gastroenterology Organization (WGO) and the American Gastroenterology Organization (13, 14). Recent studies have found that psyllium seed husk can be metabolized by gut microbiota and increase the abundance of butyric acid-producing bacteria (9). However, relevant clinical studies are limited to adult patients with chronic constipation, and there is no clinical trial on children with functional constipation.

Probiotics are live bacteria that contribute to human health after an adequate intake. They play crucial roles in maintaining the balance of the gut microbiota. Some probiotics are capable of producing butyric acid, which will maintain the integrity of the intestinal mucosal barrier by keeping the mitochondrial function of colon cells through the activation of the adenylate-activated protein kinase (AMPK) pathway (15). The damage to mitochondrial function may be related to functional dyspepsia and gastrointestinal motility disorder (16, 17). What is more, the decrease in the lactic acid-producing bacteria, such as *Lactobacillus*, might contribute to constipation, and the supplementation with *Lactobacillus* alleviates the symptoms of constipation (18, 19).

The aim of our study was to evaluate the efficacy of dietary fiber combined with probiotics on functional constipation in children with cerebral palsy. We compared the changes in gut microbiota before and after the intervention, especially the abundance of butyric acid- and lactic acid-producing bacterial genera.

## Methods

### Participant recruitment

A randomized controlled study was conducted. The study was approved by the Department of Pediatrics of Longgang District Maternity and Child Healthcare Hospital. Informed consent was obtained from all guardians of children (Ethical Approval Number: LGFYYXLLLQ-2020-002).

A total of 35 children with cerebral palsy were collected from Longgang District Social Welfare Center, all of whom experienced functional constipation and long-term bedridden and Gross Motor Function Classification System (GMFCS) level III or above. They were divided into two groups based on their daily dietary composition: 21 children with a liquid diet and 14 children with a general diet. In addition, 21 healthy children of the same age without constipation were selected as a reference group. The Nutrition Department of the Welfare Center prepared the daily diet of all the children under the supervision of a nutritionist. The liquid diet mainly consisted of milk, rice soup, and rice powder, while the general diet was primarily made up of cereals, potatoes, beans, fruits, vegetables, and a small amount of animal protein and fat. All of the participants met the diagnostic criteria for cerebral palsy, excluding those who had been diagnosed with metabolic diseases, complicated serious infections, and who had used antibiotics or probiotics within 2 weeks of intervention.

All the cerebral palsy children were administrated with Compound Dietary Fiber (CDF) powder and probiotics. CDF contained psyllium seed husk provided by BGI Precision Nutrition (Shenzhen) Technology Co., Ltd., China, which was packed as 20 g/bag. Two types of probiotic products were used in this study. One was Umeta® YiChang provided by BGI Precision Nutrition (Shenzhen) Technology Co., Ltd., China, which is administered with a dose of no <1.8 × 10^10^ CFU/sachet and consists of *Lactobacillus rhamnosus, Lactobacillus acidophilus, Lactobacillus paracasei, Lactobacillus plantarum, Bifidobacterium animalis subsp. lactis, sorbitol, fructose-oligosaccharides, and xylose*. The other one was ChangLekang® (Shandong Sinovac Biopharma Co., Ltd., China), administered at a dose of 500 mg/day and includes *Clostridium butyricum* ≥1.0 × 10^7^ CFU/g and *Bifidobacterium* ≥ 1.0 × 10^6^ CFU/g.

### Intervention

About 5 g of CDF powder was added on the 1st day for all cerebral palsy children. If no obvious GI symptoms occurred within 24 h after taking the powder, the dose should be increased by 5 g per day for those without spontaneous defecation, and the maximum daily dose should not exceed 20 g in the 1st week. In the 2nd week, the participants were given the optimal dose continuously for 1 month. At the same time, all cerebral palsy children were provided probiotics orally (1 bag/day) for 6 months. Stool samples were collected from all participants at three timepoints, including pre-intervention, 1 month, and 6 months after the intervention. The defecation frequency, stool consistency, frequency of enema used, and weight change were observed and recorded weekly, and water intake was recorded daily.

### Clinical assessment

The frequency of spontaneous defecation and manual defecation and stool consistency were used to evaluate the changes in constipation conditions in children with cerebral palsy, and body weight reflected the physical development of the children. Spontaneous defecation refers to defecation without laxative use or manual assistance, indicating the real situation of the intestinal function of participants. Manual defecation refers to those who have no spontaneous defecation for more than 72 h. Stool consistency was determined according to the Bristol stool scale (20). Stools scored as 1 or 2 on the Bristol stool scale were defined as hard, those rated 6 or 7 were defined as loose, and those rated 3, 4, or 5 were defined as normal (21).

### Sample collection

About 5 g of sample was collected from the central part of the feces and transferred to −80°C within 1 h after collection. Bacterial DNA was extracted from stool samples through PowerSoil® DNA Isolation Kit (MoBio, America), followed by the amplification of the 16s rRNA gene targeting the variable regions V3–V4. Sequencing was executed using the Illumina Miseq platform, which was performed by Novogene Co., Ltd., in China.

### Data processing and statistical analysis

High-quality data obtained after filtration and FLASH (22) were used for sequence splicing. The spliced sequences were then clustered into OTUs via USEARCH. To obtain the taxonomy profile of all samples, the representative OTU sequences were annotated to the Greengenes database (V201305) by the RDP classifier. The abundances of the bacterial taxonomy were then calculated.

According to the composition and relative abundance of all samples at the genus level, R (v3.3.3) was applied to perform the principal coordinate analysis using the ade4 package. Wilcoxon's rank-sum test was applied for comparative analysis at different taxa before and after the intervention, and Benjamini–Hochberg method was used to adjust the differences. Then the bacteria with *P*-values lower than 0.05 and FDR lower than 0.05 were regarded as significantly different ones. At the same time, the gut microbiota of the intervention (cerebral palsy children) group was compared with that of the healthy group. Spearman's Rho was performed to investigate the correlation between bacterial genera and defecation properties.

## Results

### Participant characteristics and data output

**Figure 1:**
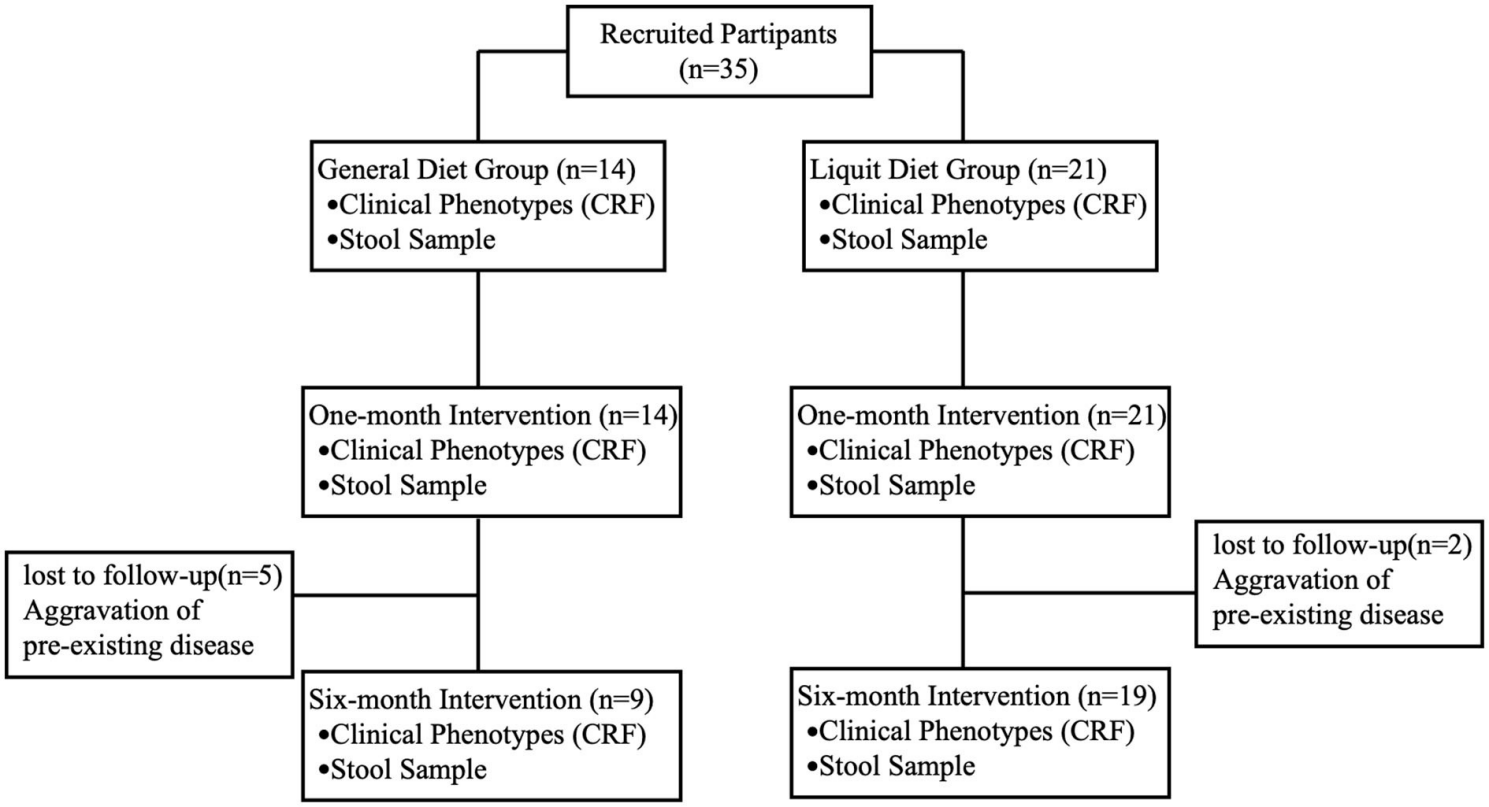
Flow chart shows the numbers and data collection of participants at each stage.

A total of 35 participants were recruited for this study, all of whom completed a 1-month intervention. Twenty-eight patients completed the 6-month intervention, including 19 in the liquid diet group and 9 in the general diet group. All seven patients (two in the liquid diet group and five in the general diet group) who lost during the follow-up were hospitalized for aggravation of the pre-existing disease, and there was no gut microbiota sequencing data of 6-month intervention for these seven patients (Figure 1). No adverse event related to any treatment was observed in this study.

The average age of the 35 children with cerebral palsy was 13.48 ± 3.40 years. The average weekly spontaneous defecation was 2.17 ± 0.57 times, and the Bristol stool score was 1.68 ± 0.47. All the children received 1–3 times manual defecation per week (1.77 ± 0.55 times per week). There were no significant differences between the two groups in baseline characteristics, including age, epilepsy, weekly defecation frequency, stool consistency, and manual defecation frequency (*P* > 0.05). Significant differences in body weight and daily water intake were observed between the two groups. The body weight of the general diet group was higher than that in the liquid diet group (*P* < 0.01) and daily water intake was less than that in the liquid diet group (*P* < 0.01) (Table 1).

**Table 1:** Demographic and clinical characteristics of enrolled children at baseline.

**Clinical phenotypes**	**All participants (*n* = 35)**	**Liquit diet group (*n* = 21)**	**General diet group (*n* = 14)**	***P*-value between groups**
Sex	19/16	9/10	4/5	0.3497
Age	13.48 ± 3.40	12.88 ± 3.09	14.38 ± 3.55	0.2242
Weight	15.75 ± 5.11	13.39 ± 3.22	19.29 ± 5.21	0.001614
Spontaneous defecation (times/week)	2.17 ± 0.57	2.29 ± 0.55	2.00 ± 0.53	0.1482
Bristol score	1.68 ± 0.47	1.71 ± 0.46	1.64 ± 0.50	0.4177
Manual defecation (times/week)	1.77 ± 0.55	1.86 ± 0.56	1.64 ± 0.48	0.2494
Water intake (mL/day)	1085.71 ± 429.87	1242.85 ± 400.71	850 ± 369.51	0.00574

A total of 105 stool samples were collected from 28 patients in two different diet groups at three timepoints, and 21 samples from healthy children were also collected. After 16S rRNA sequencing, 33002.29 ± 1843.772 tags were obtained. The total number of OTUs in the cerebral palsy group was significantly higher than that in the healthy group (*P* < 0.001). A total of 19 phyla and 285 genera were generated from all stool samples by RDP database alignment. The number of genera in patients was significantly higher than in the healthy group (*P* < 0.001).

### CDF combined with probiotics improves the constipation symptoms

After the 6-month intervention, the spontaneous defecation frequency of all the children in the cerebral palsy group increased significantly from 2.17 to 3.61 times per week (*P* < 0.0001), while manual defecation decreased from 1.77 to 0.28 times per week. The Bristol score significantly increased from 1.68 ± 0.47 to 3.71 ± 0.60 (*P* < 0.0001). The times of spontaneous defecation and Bristol score increased after 1-month intervention and then showed a decreasing trend at 6-month intervention. The times of manual defecation continued to increase from 1-month to 6-month intervention. Compared with pre-intervention, the times of spontaneous and manual defecation, and Bristol score were all significantly improved at 1-month intervention (Table 2), suggesting that CDF combined with probiotics significantly improved constipation symptoms in children with cerebral palsy.

**Table 2:** Defecation and weight in two groups at pre-, 1-month, and 6-month intervention.

**Clinical phenotypes**	**Pre-intervention**	**1-month's intervention**	**6-month's intervention**	***P* for trend**
**Spontaneous defecation**	2.17 ± 0.57	3.91 ± 0.85	3.61 ± 0.68	5.29E-14
General diet group	2.00 ± 0.55	3.71 ± 0.83	3.44 ± 0.73	1.21E-05
Liquid diet group	2.29 ± 0.56	4.05 ± 0.86	3.68 ± 0.82	7.71E-09
**Bristol score**	1.68 ± 0.47	3.80 ± 0.72	3.71 ± 0.60	1.29E-26
General diet group	1.64 ± 0.50	3.50 ± 0.52	3.89 ± 0.33	7.23E-10
Liquid diet group	1.71 ± 0.46	4.00 ± 0.77	3.63 ± 0.68	1.09E-17
**Manual defecation**	1.77 ± 0.55	0.25 ± 0.56	0.28 ± 0.60	1.62E-16
General diet group	1.64 ± 0.50	0.36 ± 0.50	0.11 ± 0.33	1.13E-06
Liquid diet group	1.86 ± 0.57	0.19 ± 0.60	0.37 ± 0.68	5.93E-11
**Weight**	15.75 ± 5.11	16.30 ± 5.16	17.51 ± 5.65	0.556
General diet group	19.29 ± 5.40	20.04 ± 5.15	23.17 ± 4.43	0.627
Liquid diet group	13.39 ± 3.30	13.81 ± 3.41	14.83 ± 3.96	0.556

The effect of dietary structure on constipation was also investigated. No significant differences were observed in spontaneous defecation frequency, manual defecation frequency, and stool consistency between the liquid diet group and the general diet group (*P* > 0.05). The times of spontaneous defecations in both groups continued to increase from 1-month to 6-month intervention (compared with pre-intervention, liquid diet group *P* (for trend) < 0.0001 and general diet group *P* (for trend) < 0.001), while the times of manual defecation was significantly declined (compared with pre-intervention, liquid diet group *P* (for trend) < 0.0001 and general diet group *P* (for trend) < 0.0001). The results demonstrated that the treatment of CDF combined with probiotics had affected constipation symptoms equally in cerebral palsy patients with a general diet and liquid diet.

### CDF combined with probiotics changed the structure of gut microbiota

**Figure 2:**
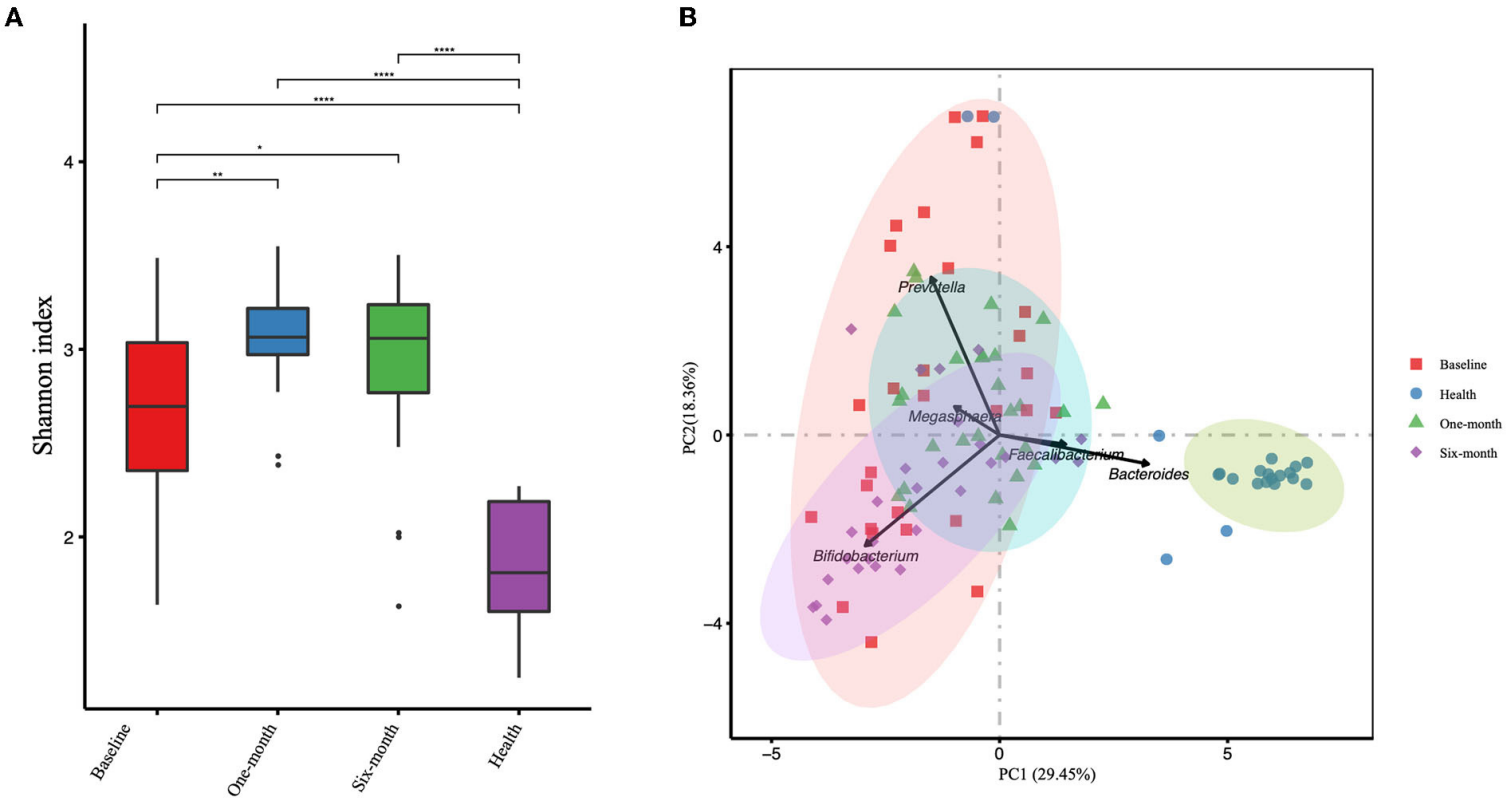
The gut microbial α-diversity analyzed by Shannon index **(A)** and the principal component analysis (PCA) **(B)** of the patient group and healthy group are presented. Three timepoints of the patient group are presented, including baseline, 1-month, and 6-month intervention. *P-value < 0.05, **P-value < 0.01, ****P-value < 0.0001.

The α-diversity was calculated based on OTUs and is represented by the Shannon index (Figure 2A). There were significant differences among four groups (including patient groups of three timepoints and healthy groups). The α-diversity in the patient groups of three timepoints was significantly higher than in the healthy group. In the patient groups, the α-diversity was significantly increased after 1-month and 6-month intervention periods (*P* = 0.0025 and *P* = 0.047, respectively). While no significant difference was observed between 1-month and 6-month intervention periods (*P* = 0.430). PCA was applied to investigate the correlation and difference between patient groups and healthy groups (Figure 2B). Stool samples of the healthy group were clustered independently and separated from the samples obtained from patients, suggesting that there were significant differences in gut microbiota structure between children with cerebral palsy and healthy children. Samples from 1-month and 6-month interventions were overlapped as well as dissociative, and both were highly co-gathered with samples from pre-intervention, which means that changes in the microbial structure could not deviate from their original state.

**Figure 3:**
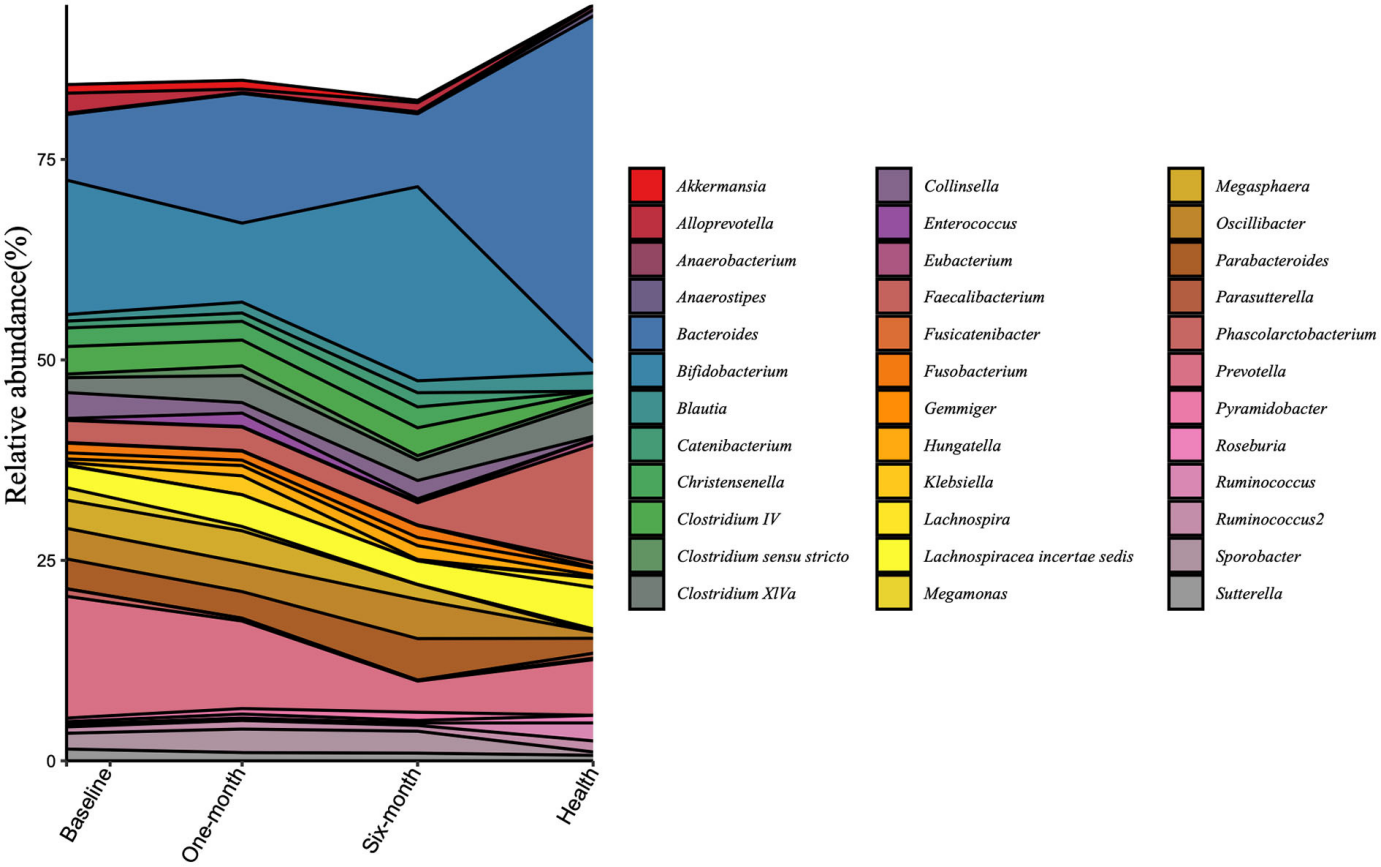
Changes in the gut microbiota at the genus level are calculated in the patient group and the healthy group at three timepoints. All the top 20 genera of each group were included.

The composition of microbiota at the genus level was analyzed in all samples (Figure 3). The top 20 bacterial genera selected from the healthy group were presented. The dominant genera were *Bifidobacterium* and *Prevotella* in patient groups, while *Bacteroides* and *Faecalibacterium* dominated in the healthy group. There were various trends in relative abundances of genera before and after the intervention. *Prevotella, Collinsella, Sutterella*, and *Megamonas* consistently declined after the intervention. As for *Bacteroides, Faecalibacterium*, and *Lachnospiracea incertae sedis*, although they went upward at the 1-month visit, they all suffered from a downward trend at the 6-month visit. In contrast, there were opposite trends, falling to the lowest points and then rising again, such as *Bifidobacterium, Oscillibacter*, and *Parabacteroides*.

### Gut microbiota was influenced by diet structure

**Figure 4:**
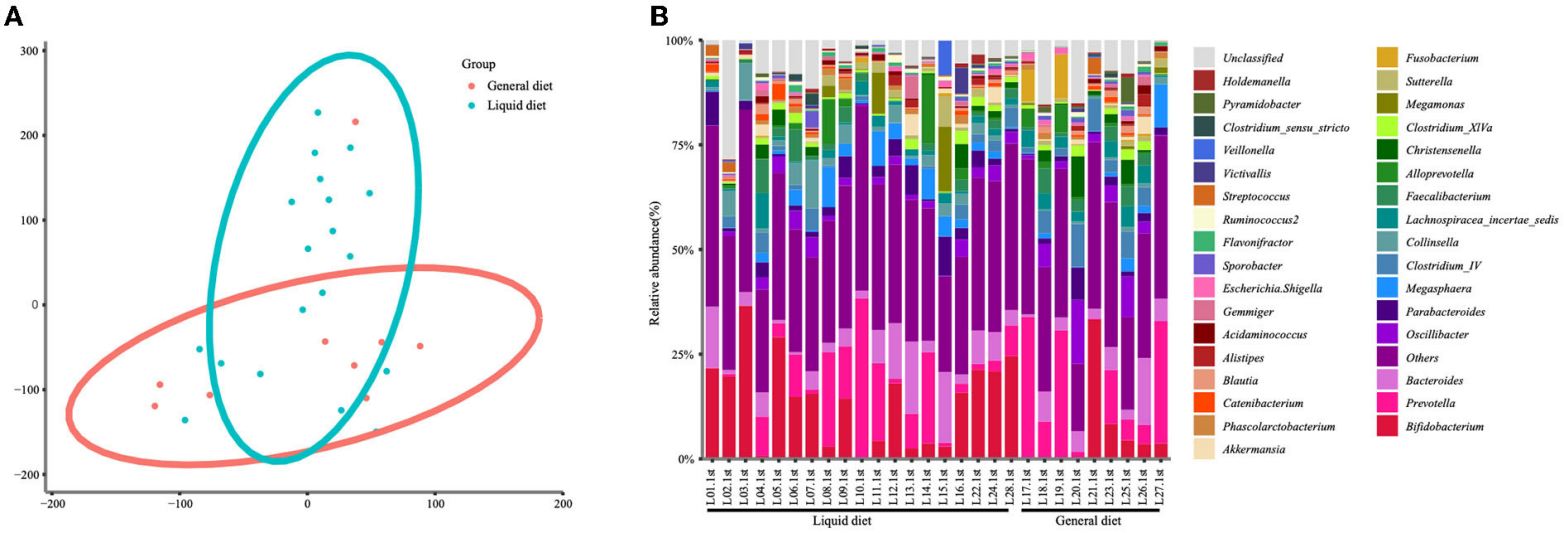
Changes in gut microbiota at the genus level in the children of the general diet group and liquid diet group. The clustering diagram of the two groups **(A)** and the stacked bars of the top 30 abundant genera of each participant are shown **(B)**.

Considering that diet is a crucial factor that shaped gut microbiota, the gut microbiota was also analyzed based on two diet groups: the liquid diet group and the general diet group. The PCA showed significant differences between the two groups (Figure 4A). There were statistical differences between general diet group and liquid diet group in the following genera: *Bifidobacterium* (8.82 vs. 20.68%, *P* = 0.0344, FDR = 0.03789), *Clostridium* IV (5.71 vs. 2.21%, *P* = 0.0304, FDR = 0.3789), *Fusobacterium* (3.27 vs. 0.23%, *P* = 0.0304, FDR = 0.3789), and *Collinsella* (0.89 vs. 4.34%, *P* = 0.0027, FDR = 0.3789). Gut microbiota in the general diet group was dominated by *Prevotella*, while that in the liquid diet group was dominated by *Bifidobacterium* (Figure 4B, Supplementary Table 1).

**Figure 5:**
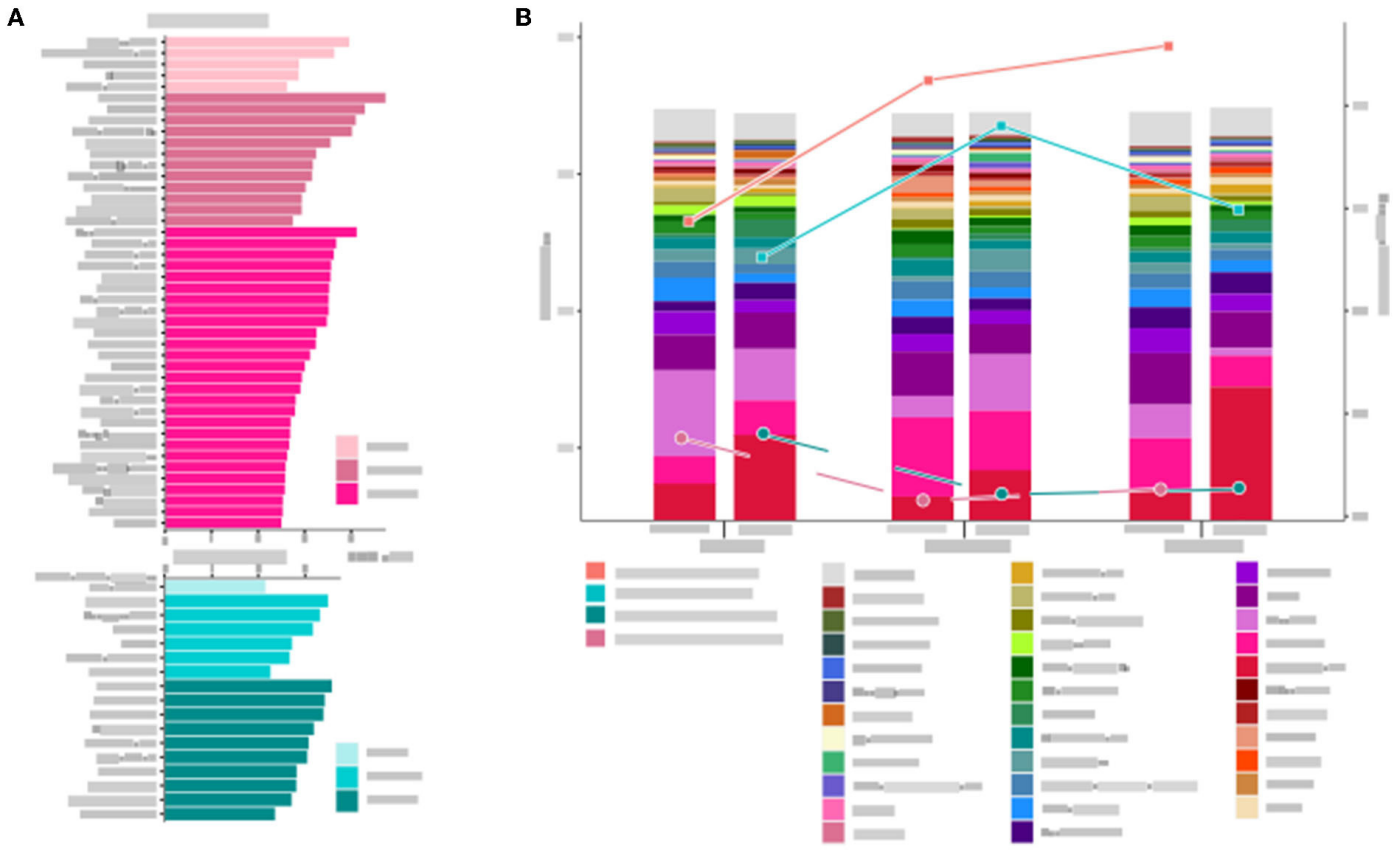
Microbial structure and diversity of the gut microbiota in the general diet group and liquid diet group at three timepoints are analyzed. **(A)** Changes in genera at three timepoints. **(B)** The stacked bars show microbial composition at the genus level, and the trend of α- and β- diversity at three timepoints are presented by Shannon index and Brat–Curtis distance, respectively.

The number of bacterial genera gradually increased from 1 month to 6 months after the intervention in the two groups (Figure 5A). The α-diversity in the general diet group was higher than that in the liquid diet group at all three timepoints of intervention. The α-diversity in the general diet group kept increasing, while that in the liquid diet group increased first and then decreased, but was still higher than the baseline value at the end of the intervention period. The Bray–Curtis distance between the general diet and liquid diet groups gradually narrowed during the 6-month intervention (Figure 5B). It suggested that there was a sustained response in gut microbiota in the general diet group, while inadequate response was presented in the liquid diet group. Therefore, longer intervention time should be considered in future studies.

The top 20 abundant genera were selected to investigate the changes in the microbiota structure in two groups. In the general diet group, most genera responded significantly after 1-month intervention and responded weakly at 6-month intervention. In the liquid diet group, the abundance of most genera increased or decreased after 1-month intervention, and then reversed changes were observed after 6 months of intervention. For example, *Bifidobacterium* decreased at 1-month intervention and then increased at 6-month intervention, whereas *Prevotella* and *Bacteroides* elevated at 1-month intervention and then decreased at 6-month intervention. A few genera showed an increasing or a decreasing trend. The changes in relative abundance showed four patterns in the two groups: rise first and then decline again (*Bacteroides, Lachnospiracea incertae sedis, Faecalibacterium, Clostridium XlVa, Blautia, Escherichia/Shigella, Klebsiella, Akkermansia*, and *Streptococcus* in the general diet group; *Prevotella, Bacteroides, Faecalibacterium, Lachnospiracea incerta sedis, Clostridium XlVa, Akkermansia*, and *Blautia* in the liquid diet group), decline first and then rise (*Prevotella, Bifidobacterium, Clostridium IV, Oscillibacter, Fusobacterium, Megasphaera, Alloprevotella*, and *Collinsella* in the general diet group; *Bifidobacterium, Collinsella, Parabacteroides, Megasphaera, Alloprevotella, Megamonas, Sutterella, Gemmiger*, and *Alistipes* in the liquid diet group), sustained increase (*Parabacteroides* in general diet group; *Oscillibacter, Clostridium IV*, and *Catenibacterium* in liquid diet group), and sustained decrease (*Sutterella* in the general diet group; *Phascolarctobacterium* in the liquid diet group).

### Probiotic supplements elevated the abundance of *Lactobacillus* and *Clostridium*

In order to evaluate the survival ability of the supplied probiotic, including *Bifidobacterium, Lactobacillus*, and *Clostridium butyricum*, in the GI tract after oral administration, their abundance changes in the two groups of children after intervention were investigated.

*Lactobacillus* and *Clostridium* consistently increased both in general diet and liquid groups during the 6-month intervention, suggesting that the supplemented probiotics may colonize in the gut. However, *Bifidobacterium* decreased at the 1-month visit and then increased at the 6-month visit, but no statistical differences were observed between the general diet and liquid diet groups (Supplementary Table 4). This finding indicated that the exogenous *Bifidobacterium* showed less ability to colonize the gut than *Lactobacillus* and *Clostridium* in children with constipation.

### Clinical phenotypes were correlated with gut microbiota

**Figure 6:**
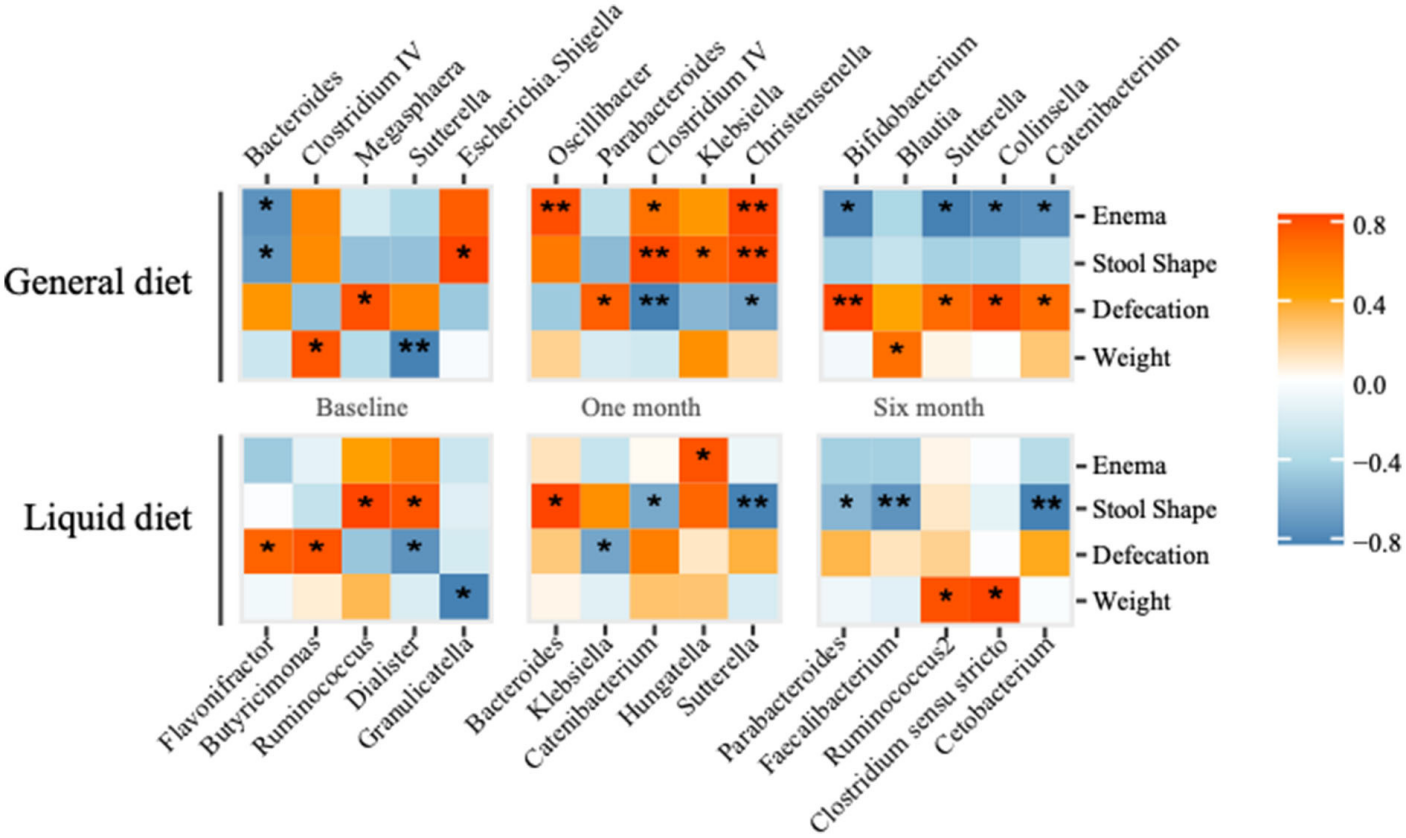
Correlation analysis between gut microbiota (top five abundant genera) and clinical phonotypes in general diet and liquid diet groups are calculated. *P-value < 0.05, **P-value < 0.01.

The correlation analysis was applied to investigate the relationship between gut microbiota and clinical phenotypes, such as body weight, defecation, stool shape, and the need for an enema. In the general diet group, there was a negative correlation between body weight and abundance of *Sutterella*, while positively correlated with *Clostridium IV* at baseline (Figure 6). After the 1-month intervention, manual defecation was positively related to *Oscillibacter* and *Christensenella*, and fecal shape was positively correlated with *Clostridium IV* and *Christensenella*, while the defecation smoothness was negatively correlated with *Clostridium IV*. Therefore, defecation smoothness was positively correlated with *Bifidobacterium* after the 6-month intervention. While in the liquid diet group, the fecal shape was negatively related to *Sutterella* after the 1-month intervention and negatively correlated with *Faecalibacterium* and *Catenibacterium* after the 6-month intervention.

## Discussion

Accurate supplementation of dietary fiber or probiotics based on gut microbiota characteristics can effectively improve functional constipation in children with cerebral palsy. In this study, supplementation with dietary fiber and probiotics resulted in a significant improvement in defecation frequency and water content of feces after a 1-month intervention, especially in the liquid diet group. This may be related to the raised abundance of *Lactobacillus* and butyrate-producing genera, which optimized the gut microbiota in children and therefore promoted colonic propulsive peristalsis. However, after short-term dietary fiber and long-term oral probiotics intervention, there were still two patients in the liquid diet group who experienced recurrent constipation and obviously required manual defecation, and the abundance of *Prevotella, Oscillibacter, Bacteroides*, and *Bifidobacterium* in their gut microbiota also rebounded, suggesting that the intervention time of dietary fiber is too short and supplement of probiotic alone fails to achieve the expected effect. Moreover, the loss of the live bacteria in the probiotic product may lead to lower bacterial colonization in the gut and further influence the outcome of the trial. Therefore, further research on the bacterial stability in the probiotic product is required. Moreover, insufficient course of dietary fiber and too little dietary carbohydrate intake may also be responsible for the higher recurrent rate in the liquid diet group.

There were significant differences in the abundance of bacterial genera between the liquid diet group and the general diet group at baseline, including *Fusobacterium, Alloprevotella, Bifidobacterium, Lachnospiracea incertae*, and *Collinsella*, which were similar to our previous study (23). Varied abundance of *Fusobacterium* and *Alloprevotella*, which could cause oral diseases such as periodontitis (24, 25) and intestinal inflammation (26, 27), was observed in the two groups of children. Consumption of milk powder was responsible for the high abundance of *Bifidobacterium* in the liquid diet group (28). Compared with the general diet group, we can see a higher abundance of opportunistic pathogen *Collinsella* and lower butyrate-producing genus *Lachnospiracea incertae* in the liquid diet group, which can induce severe intestinal inflammation (19, 29). The results reflect that the imbalance in the gut microbiota of children in the liquid group leads to more severe GI dysfunction, which is consistent with the incidence of constipation in the liquid group in this study and our previous study (30).

Significant changes in the gut microbiota between pre- and post-interventions were discovered in two groups. Particularly, after 1-month intervention, the abundance of butyrate-producing bacteria like *Bacteroides, Lachnospiracea incertae sedis, Faecalibacterium*, and *Clostridium XlVa* experienced a pronounced increase, while that of opportunistic pathogens like *Alloprevotella, Megasphaera*, and *Collinsella* declined. However, an opposite pattern occurred after the 6-month intervention. The abundance of butyrate-producing genera decreased, while the abundance of opportunistic pathogens rebounded. It was found that both groups showed an increasing trend in α-diversity after the 1-month intervention. Although this trend was more obvious in the liquid diet group, the trend reversed after the 6-month intervention, which may be related to the significant increase in the abundance of *Bifidobacterium* due to the intake of milk powder. In contrast, no significant changes were observed in *Bifidobacterium* in the general diet group before and after the intervention. The Bray–Curtis distance decreased after the intervention, indicating that the intervention could reduce the differences in the gut microbiota between the two groups, which was significantly correlated with the improvement in clinical constipation and was also in line with our expected goal. However, current sequencing technology can only explain the composition of the microbiota. Direct evidence of microbial metabolites and functions associated with the gut microbiome and their role in the progression of constipation in cerebral palsy children still needs further studies (31).

The correlation analysis between clinical phenotypes and gut microbiota in children with cerebral palsy showed that the changes in the abundance of some bacteria in two groups (liquid diet and general diet group) correlated with constipation symptoms, the times of defecation, and the enema demand. In this study, defecation, stool shape, and enema were significantly correlated with *Sutterella, Oscillibacter, Clostridium IV, Christensenella, Bifidobacterium, Faecalibacterium*, and *Catenibacterium*. It was reported that *Sutterella* can cause digestive disorders (15), and *Oscillibacter* is associated with ulcerative colitis (32). In contrast, *Clostridium IV, Bifidobacterium*, and *Faecalibacterium* are beneficial bacteria in the human gut, which can protect the integrity of intestinal mucosa through metabolites such as short-chain fatty acids (33, 34). Based on correlation analysis, supplementation of butyrate-producing bacteria can reduce intestinal inflammation and relieve GI dysfunction in children with cerebral palsy.

In the current study, we also found that the intervention of diary fiber and probiotics increased the abundance of beneficial bacteria in the gut microbiota, such as *Lactobacillus* and *Bifidobacterium*. However, the abundance of *Bifidobacterium* decreased before increasing. *Bifidobacterium* is the dominant genus in the gut microbiota of infants, and its abundance will decrease along with the maturity of gut microbiota. The abundance of *Bifidobacterium* significantly decreased and that of butyrate-producing bacteria increased after the intake of dietary fiber combined with probiotics, indicating that this intervention method may be beneficial to the maturity of children's gut microbiota. Moreover, in the subsequent probiotic intervention, the abundance of *Bifidobacterium* rose again, indicating that the effect of probiotic intervention alone may be insufficient when compared to combined intervention. Further randomized controlled studies are still needed to prove the independent effect of each product.

The purpose of this study was to solve practical clinical problems. The lack of a control group and relatively small sample size may not reflect the accurate outcomes in the real world. Due to the lack of the previous exploration of intervention dose and course of treatment, the children received probiotics for 6 months but CDF for only 1 month, resulting in recurrent constipation cases in the liquid diet group, which should be improved in the future study. We speculated that if the intervention time of dietary fiber and probiotics is prolonged, constipation in children would show long-term remission, which would further promote the absorption and metabolism of nutrients, improve immunity, and further contribute to the recovery of brain function.

## Conclusion

Based on the characteristics of gut microbiota in children with cerebral palsy, different diet structures influence the composition of gut microbiota. Long-term consumption of a liquid diet causes an imbalance in the gut microbiota and thus leads to GI dysfunction in children with cerebral palsy. The supplement of CDF and probiotics can effectively improve this situation, which significantly increased the abundance of protective bacteria and lowered the abundance of symbiotic pathogens. Simultaneously, supplementation of CDF combined with probiotics can improve functional constipation in children with cerebral palsy.

## Data availability statement

The data presented in the study are deposited in the NCBI SRA database repository, accession number PRJNA881289.

## Ethics statement

The studies involving human participants were reviewed and approved by Department of Pediatrics of Longgang District Maternity and Child Healthcare Hospital. Written informed consent to participate in this study was provided by the participants' legal guardian/next of kin. This study was approved by the Ethics Committee of Longgang District Maternity and Child Healthcare Hospital of Shenzhen city with the registration number of LGFYYXLL-024.

## Author contributions

CH and CC conceived the project. CH recruited the participants and performed the sampling. CC assisted the sample collection, responsible for the sample transportation and preservation, as well as the basic information collection, and organized the discussion. LG was responsible for the process of project and participants' follow-up. JL, YP, and ZY performed data analysis. SX, BW, and XC contributed to patients recruiting and information collection. CH, JL, CC, and XZ accomplished the manuscript, among which CH and JL drafted the manuscript. XZ revised the manuscript and responsible for the whole project. All authors contributed to the article and approved the submitted version.

## Funding

This work has been strongly supported by Longgang District Science and Technology Innovation Bureau (LGKCYLWS2020104) and National Natural Science Foundation of China (82201793).

## Conflict of interest

Author LG was employed by the company BGI Nutrition Precision Co., Ltd. Author ZY was employed by the company Shenzhen WeHealthGene Co., Ltd. The remaining authors declare that the research was conducted in the absence of any commercial or financial relationships that could be construed as a potential conflict of interest.

## Publisher's note

All claims expressed in this article are solely those of the authors and do not necessarily represent those of their affiliated organizations, or those of the publisher, the editors and the reviewers. Any product that may be evaluated in this article, or claim that may be made by its manufacturer, is not guaranteed or endorsed by the publisher.
