# Corrigendum: Dietary fiber and probiotics based on gut microbiota targeting for functional constipation in children with cerebral palsy

**DOI:** 10.3389/fped.2022.1074856

**Published:** 2022-11-21

**Authors:** Congfu Huang, Jinli Lyu, Chunuo Chu, Lan Ge, Yuanping Peng, Zhenyu Yang, Shenghua Xiong, Bin Wu, Xiao Chen, Xiaowei Zhang

**Affiliations:** ^1^ Department of Pediatrics, Longgang District Maternity and Child Healthcare Hospital, Shenzhen, China; ^2^ Department of Obstetrics and Gynecology, Peking University Shenzhen Hospital, Shenzhen, China; ^3^ Shenzhen Middle School, Shenzhen, China; ^4^ Department of Nutrition, BGI Nutrition Precision Co., Ltd., Shenzhen, China; ^5^ The Outpatient Department, Longgang District Social Welfare Center, Shenzhen, China; ^6^ Department of Microbial Research, WeHealthGene Institute, Joint Laboratory of Micro-Ecology and Children’s Health, Shenzhen Children’s Hospital, Shenzhen WeHealthGene Co., Ltd., Shenzhen, China

**Keywords:** gut microbiota, dietary fiber, probiotics, cerebral palsy, functional constipation

A Corrigendum on Dietary fiber and probiotics based on gut microbiota targeting for functional constipation in children with cerebral palsy By Huang C, Lyu J, Chu C, Ge L, Peng Y, Yang Z, Xiong S, Wu B, Chen X and Zhang X. (2022) Front. Pediatr. 10: 1001789. doi: 10.3389/fped.2022.1001789

In the published article, author Chunuo Chu was omitted from co-first authorship. The corrected author list appears below.

Congfu Huang^1^†, Jinli Lyu^2^†, Chunuo Chu^3^†, Lan Ge^4^, Yuanping Peng^5^, Zhenyu Yang^6^, Shenghua Xiong^1^, Bin Wu^1^, Xiao Chen^1^ and Xiaowei Zhang^2*^.

The authors apologize for this error and state that this does not change the scientific conclusions of the article in any way. The original article has been updated.

